# The Metals in the Biological Periodic System of the Elements: Concepts and Conjectures

**DOI:** 10.3390/ijms17010066

**Published:** 2016-01-05

**Authors:** Wolfgang Maret

**Affiliations:** London Iron Metabolism Group, Division of Diabetes and Nutritional Sciences and Department of Biochemistry, Faculty of Life Sciences and Medicine, King’s College London, 150 Stamford St., London SE1 9NH, UK; wolfgang.maret@kcl.ac.uk; Tel.: +44-20-7848-4264; Fax: +44-20-7848-4195

**Keywords:** essential metals, non-essential metals, periodic system of the elements, metallomics

## Abstract

A significant number of chemical elements are either essential for life with known functions, or present in organisms with poorly defined functional outcomes. We do not know all the essential elements with certainty and we know even less about the functions of apparently non-essential elements. In this article, I discuss a basis for a biological periodic system of the elements and that biochemistry should include the elements that are traditionally part of inorganic chemistry and not only those that are in the purview of organic chemistry. A biological periodic system of the elements needs to specify what “essential” means and to which biological species it refers. It represents a snapshot of our present knowledge and is expected to undergo further modifications in the future. An integrated approach of biometal sciences called metallomics is required to understand the interactions of metal ions, the biological functions that their chemical structures acquire in the biological system, and how their usage is fine-tuned in biological species and in populations of species with genetic variations (the variome).

## 1. Introduction

**Figure 1:**
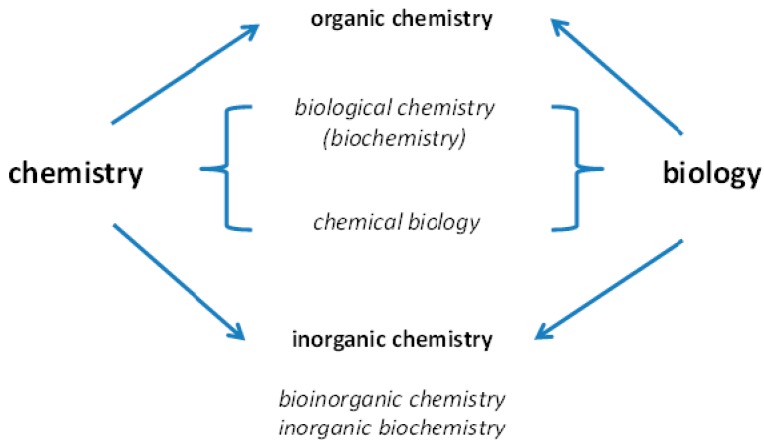
Both chemistry and biology come to bear on investigations of the roles of chemical elements in organisms.

Many scientific disciplines address which chemical elements are present in organisms and which functions they have. Chemistry and biology are contributing to such investigations. With time, more specialized disciplines developed with emphasis on either organic or inorganic chemistry (Figure 1).

The names of the sub-disciplines can be misleading when organic chemistry is associated with life and inorganic chemistry with the inanimate world only. Life is built on organic as well as inorganic compounds, and, as I shall discuss in this article, significant more chemical elements than the ones commonly treated in organic chemistry are important for life. Among them are many metal ions that have contributed critically to the evolution of life—life would not be possible without them. Metallomics, an integrated biometal science, aims to serve as a discipline that covers all aspects of how metals function in biological systems.

One can distinguish three developments that led to our present knowledge, all connected to a shift in the types of questions addressed and all on-going.

First, attempts were made to determine which elements occur in living organisms and which ones are essential for life. In a 70 kg human, the bulk elements, hydrogen (H), carbon (C), nitrogen (N), oxygen (O), and sulphur (S) are over 1 kg, except for sulphur, which is only about 100 g. Macrominerals were identified, for instance the metal cations of sodium (Na), potassium (K), magnesium (Mg), and calcium (Ca) and the non-metals phosphorous (P) and chlorine (Cl), which are in the gram range except for calcium, which is about 1.7 kg. Trace element research matured with advances in analytical chemistry and instrumentation and provided evidence for additional essential elements, the metal ions of iron (Fe), zinc (Zn), manganese (Mn), copper (Cu), cobalt (Co) in the form of vitamin B_12_, molybdenum (Mo) and the non-metals selenium (Se) and iodine (I). They are in the mg range, except for iron and zinc which are 2–5 g and thus strictly speaking not trace elements. Yet other elements are present at even lower levels but noteworthy some are present above the levels of essential elements such as Mn and Mo, e.g., bromine (Br), rubidium (Rb), aluminium (Al), nickel (Ni), titanium (Ti) and barium (Ba). 

Second, we witnessed the isolation of metalloproteins and the characterization of their coordination environments in which the metal ions function. Bioinformatics allowed for estimates that about 50% of all enzymes depend on a metal ion for catalysis, demonstrating the significance of metal ions in biochemistry. For zinc alone, about 3000 human proteins have the structural features to be zinc metalloproteins, meaning that about every tenth protein encoded in the human genome contains zinc [1]. Knowledge about interactions of metals with biological macromolecules other than proteins *in vivo* is scarce. An exception is magnesium, which has a role in RNA structure and function. 

Third, proteins with specific roles in metal metabolism such as membrane metal transporters, metalloregulators, and metallochaperones were characterized. These discoveries brought insight into the complex biological mechanisms of metal regulation and selectivity. It turned out that each metal ion is controlled in a characteristic range of concentrations that is determined by the affinities of metal ions for their ligands. Thus, in the series Mg, Ca, Mn, Fe, Co, Ni, Zn, Cu, the binding strength (affinity) increases and accordingly, the free metal ion concentrations-metal ions not bound to proteins-decreases. In principle, this series conforms to the Irving-Williams series for divalent metal ions [2]. Although Mg complexes are usually stronger than Ca complexes, Ca is stabilized over Mg in biology by using additional ligands and thus coordination numbers higher than six. This stabilization is necessary because Ca^2+^ has been adopted as a cellular signalling ion controlling many processes. Zn is positioned after Cu in the periodic system of the elements (PSE), but complexes of Cu have higher stability. In the cell, Cu is the only metal ion in this series that is primarily monovalent (Cu(I)). A most remarkable consequence of these ranges of free metal ion concentrations is that they cover about 15 orders of magnitude, from millimolar (Mg) to attomolar (Cu). Thus metal ions can be used in biological processes over an extremely wide range, demonstrating in essence the enormous contribution that metal ions make to biological function. Total cellular metal ion concentrations in humans, however, follow a different series: Mg, Ca, Fe, Zn, Cu, Mn, Co. Hence, to maintain the trend expressed by the Irving-Williams series for characteristic free metal ion concentrations, biology has to adjust for the rather large differences in total metal concentrations. It also has to deal with the fluctuations/oscillations of signalling metal ions. In addition to Ca^2+^, Zn^2+^ is also a cellular signalling ion. Whereas Ca^2+^ activates many processes, Zn^2+^ seems to be primarily an inhibitory ion and due to its preference for different ligands and different coordination environments it targets other sets of proteins [3].

## 2. The Essential Elements

Considering our vast knowledge about genes and proteins, it is quite remarkable that our knowledge about the role of chemical elements in life remains limited and open-ended. Only last year, bromine was added to the list of essential elements with its function in collagen metabolism [4]. Hence we should not assume that we know all the elements that are essential for animals and humans as uncertainties about the biochemical functions of some chemical elements persist. Striking as this statement may appear, the lack of knowledge provides ample opportunities for discoveries with significant implications for biochemistry and improving health. 

We now know that 11 metals and 10 non-metals, *i.e.*, 21 elements, are essential for humans. The count includes chromium whose status as an essential element is controversial (see below). Additional elements are essential for other forms of life outside the kingdom animalia. Therefore when essential elements are listed in a “biological periodic system of the elements (PSE)” the meaning and implications usually are not clear. Conjectures arise mainly because of two issues: 

“Essential” should include a reference to the biological species. Biological PSEs are usually presented with all the elements identified as essential in all species. However, some elements are essential to only some organisms. I propose to categorize in the following way: (i) elements that are essential to all species; (ii) elements that are used in a significant number of species (V, Ni); and (iii) elements that are used only in a few organisms in special ecological niches (W, Cd, Lanthanides). Vanadium is used in some nitrogenases and haloperoxidases in algae and fungi. Nickel has been discovered in only nine enzymes in some bacteria and plants. Tungsten is used instead of molybdenum in enzymes of some thermophilic bacteria. Ni, V, W are not established as being essential for animals and humans. Orthologous gene products requiring these elements have not been found in animals and humans. Carbonic anhydrase, which is usually a zinc enzyme, is a cadmium enzyme in the marine diatom *Thalassiosira weissflogii* [5]. The US environmental protection agency (EPA) has classified cadmium as a Group B1 probable human carcinogen. Lanthanides (rare earth elements, REE) instead of calcium were found as cofactors of methanol dehydrogenase of particular methanotrophic bacteria [6]. While this wider usage of metal ions in life is interesting from the standpoint of evolution and certainly revealed fascinating chemistries, the utilization of specific elements such as Cd and REE, is of limited significance for nutrition of animals and humans. 

“Essential” should include a definition. Elements that are essential for survival should be distinguished from others that have limited functions and some health benefits only. An example is fluorine which as fluoride prevents dental caries and perhaps is beneficial for bone health but otherwise exhibits toxicity. A broader definition had been introduced: “An element is essential when a deficient intake consistently results in an impairment of a function from optimal to suboptimal and when supplementation with physiological levels of this element, but not others, prevents or cures this impairment” [7]. Following this definition, additional elements were classified as essential, but they are not necessarily essential for life [8]. Several of them we know to be beneficial for animals and humans, e.g., B, Cr, Ni, Si, and accordingly, there is an on-going discussion whether guidelines for their intake should be given [9,10,11]. 

Biological chromium research illustrates the difficulties in defining structure and function of biologically active metal ions. Chromium is an example of the importance of chemical speciation in biology: Chromium(III) complexes are the ones having beneficial functions and little toxicity, whereas Cr(VI), chromate, is a human carcinogen. Chromium(III) deserves further discussion as it is widely accepted as an essential trace metal and accordingly dietary reference intakes (DRIs) were issued and it is available as a dietary supplement [12]. However, its status as an essential element was questioned recently [13]. After sixty years of chromium research some crucial questions remain unanswered: (i) the structure of a biologically active chromium complex; (ii) ways to determine chromium status in humans; and (iii) elucidation of its exact mechanism of action in glucose and lipid metabolism [14]. One should concede that not having succeeded in isolating a biologically active chromium complex would seem not to be an issue: absence of evidence is not evidence for absence. The low amount of chromium per se should also not be a concern as molybdenum and cobalt occur at equally low concentrations.

**Figure 2:**
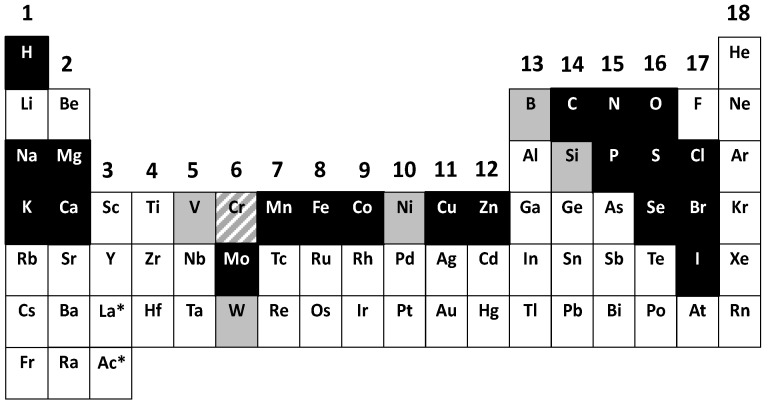
A biological periodic system of the elements (PSE) indicating the essential elements. The essential elements for most forms of life are shown in black with the exception of chromium (Cr), which is shown with an upward diagonal pattern (see text), and the essential elements that are more restricted for some forms of life are shown in grey. Not shown are the f-group elements: lanthanides and actinides (asterisk after lanthanum (La) and actinium (Ac). The groups are numbered 1–18.

A biological PSE that considers the two issues associated with the meaning of “essential” and separates general from specific cases (Figure 2) demonstrates that biochemistry, the chemistry of life, depends on a significant number of elements. The main features of such a PSE are that (i) most of the non-metals, forming a triangle in the upper right part of the PSE, are used (except the noble gases); (ii) from the metalloids (B, Si, Ge, As, Sb, Te) only B and Si are known to have beneficial effects for humans and are essential for some species; and (iii) the entirety of group 13 with the exception of boron remains unused. The individual elements in a biological periodic table have been discussed in detail [15]. 

Given the heightened interest in the fascinating and challenging chemistry of some metals in special microbes, it is instructive to recall the relative significance of the transition metals and zinc in mammalian biology: Fe ≈ Zn > Cu > Mn > Mo (in the pterin cofactor) and Co (in the corrin cofactor). A 70 kg human contains about 5 g Fe, 2 g Zn, 100 mg Cu, 12–20 mg Mn, 5 mg Mo, and 2 mg Cr and Co.

Mining genome database for signatures of metal binding sites in protein sequences allowed for estimates of the number of metalloproteins containing zinc, iron, and copper. With such predictions a true understanding of these three metals at the systems level, *i.e.*, their metalloproteomes, is emerging. Efforts are under way to annotate functions of these metalloproteins on the basis of similarities with structures of proteins with known functions. In humans, there are only four known molybdoenzymes and only two vitamin B_12_-dependent enzymes. However, estimates of the number of enzymes that use manganese as a cofactor are lacking. 

It seems that we do not even have a complete list of essential elements for one given species. The identification of roles of elements such as Cd and REE in metalloproteins (see above) indicates that our knowledge about essentiality of chemical elements in different forms of life is incomplete. An “omics” study also came to the conclusion that metalloproteomes of microorganisms are largely uncharacterized [16]. And then there is the issue what functions the other elements present have.

## 3. The Non-Essential Elements

**Figure 3:**
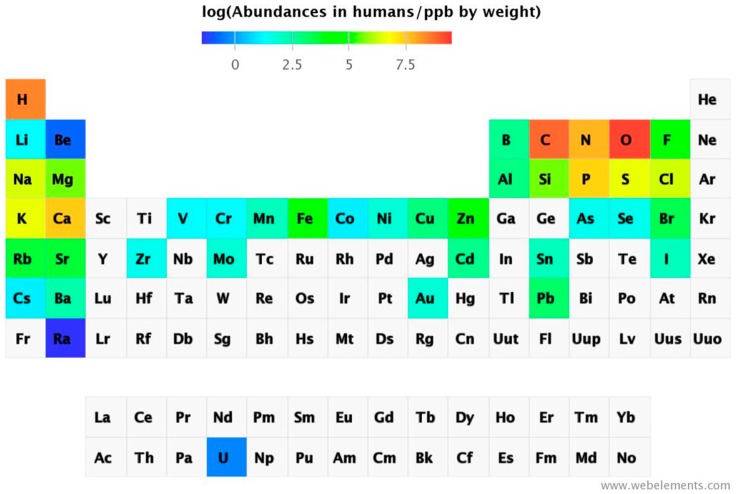
Abundance of the chemical elements in humans [18].

Instrumental analytical chemistry for metal determination has advanced significantly. Inductively-coupled plasma mass spectrometry (ICP-MS) has sub-ppt detection limits, allowing for detection of virtually all natural occurring elements in biological samples. Even radioactive uranium can be detected. Thus, 74 out of 78 elements were found in salmon eggs [17]. The abundance of chemical elements in humans has been summarized in a periodic table (Figure 3).

Some non-essential elements are present at significantly higher concentrations than the essential ones present at the lowest concentration. Sr is present at ppm levels, and Rb, Ba, Ni, As, Al, and Ti (not shown in Figure 3) at ppb levels, but also others that are considered to have mainly toxic actions, e.g., Cd, Hg (not shown in Figure 3), and Pb are present, but they are variable and depend on exposure. Some elements can accumulate with age, while others are rather tightly regulated. Distribution of concentrations has been suggested as a criterion for elements being essential, namely homeostatic control for essential elements leading to a normal distribution but the presence of non-essential elements following a skewed distribution depending on exposure [8]. While some elements are bioactive with positive effects on health, many non-essential elements nevertheless are bioreactive [16]. They are not chemically inert, and can serve as catalysts, be pharmacologically active, or toxic. Therefore, it is important to adopt a broader view of metal ions in biology, beyond a focus on essential metal ions, to include the many elements that are present with poorly understood functional consequences. We know very little how and to which extent animals and humans can cope with these apparently non-essential elements, whether the presence of some reflects a lack of selectivity in uptake mechanisms, whether there are any specific mechanisms of detoxification, and what the exposure limits and biological effects are. 

The delicate balance among the essential elements extends to complex interactions with non-essential elements and other nutrients. Too much (overload) or too little (deficiency) of one has conditioning effects on others. For example, zinc supplementation can cause copper deficiency [19]. Deficiency of an essential element can lead to the uptake of a non-essential element with potential toxicity. For instance, under iron deficiency, uptake of cadmium increases and interferes with the metabolism of essential elements, such as zinc. 

A focus on essential elements only is not sufficient. We need to monitor the presence of non-essential elements, how their concentrations vary and how they affect functions of the biological system. For the essential elements, we have some understanding of how deficiencies or overload perturb functions though cause and effect in chronic diseases is often not clear. For the non-essential elements effects at sub-toxic concentrations are more difficult to assess. For example, in 2012 the Centers of Disease Control and Prevention lowered blood lead levels of concern from 10 µg/dL to ≥5 µg/dL for children aged 1–5 to identify children and environments with lead-exposure hazards because neurobiological parameters are affected negatively even at previously accepted blood lead levels [20]. Average normal levels in the US population are 1.6 µg/dL for adults and 1.9 µg/dL for children. Clearly, we need additional solid data how each individual element affects animals and humans. 

We also are beginning to realize that there are a significant number of inherited diseases of human metal metabolism. Comparatively fewer variations have been documented in animals. Our knowledge is very limited on how mutations in the many genes that encode metal regulatory processes affect the toxicity of metals, and metal dose-responses quantitatively. Polymorphisms in the about 12 human metallothionein genes suggest an effect on metal metabolism [21]. For example, an Asn27Thr substitution in metallothionein-1A affects its zinc-binding capacity and together with polymorphisms of other human metallothionein genes is associated with the risk of developing type 2 diabetes, coronary heart disease, and other complications of diabetes [22,23] and with altered metabolism of toxic metal ions, *i.e.*, cadmium, lead, and mercury [24,25,26]. Levels for toxic metals in blood are associated with polymorphisms in metal transporter genes and other genes, thus providing further evidence that subsets of individuals are more susceptible to the toxic effects of some metal ions [27].

New applications and manufacturing processes expose animals and humans increasingly to a number of metals to which they have not been exposed to in the past, in particular those from the bottom part of the PSE. Food chains and food webs amplify some exposures, which has largely unexplored effects for more recently employed metal ions. REE find applications in batteries, magnets, lighting, optical fibers; gallium, indium, and tellurium in solar cells; hafnium and tantalum in optical devices, and elements from the platinum group ruthenium, rhodium, palladium and their analogues osmium, iridium and platinum are used in fuel cells [28]. Another issue is the increasing applications for nanomaterials. They have different and often enhanced reactivities. Also, we are exposed to metallodrugs for therapeutic or diagnostic purposes, and use implants (Si), metal prostheses (Co), and dental fillings (Hg) with functional consequences for living systems. The increased use of additional elements adds a new dimension to exposure and pollution in air, soil, and water with fundamental implications for health and understanding etiology and pathology. 

Most organisms live in a symbiotic relationship with others and need to defend themselves against parasites. The gut harbours a huge number of commensal bacteria (the microbiome) that are involved in processing food. An intriguing aspect is that microorganisms rely on a different complement of metal ions and utilize metal ions that animals and humans apparently do not use. For example, many bacteria need nickel, including the ulcer-causing pathogen *Helicobacter pyloris*. Hence depriving this organism of nickel provides a selective therapeutic means for its eradication in the upper digestive tract. Some of the trace element deficiencies observed could be due to an effect on symbiotic organisms that require particular trace elements, rather than on the host directly.

## 4. Conclusions

Chemistry and biology must come together in investigations of biometals. Neither establishing only structures nor finding only functions is sufficient. For example, sugars and lipids incorporate arsenic, but the characterization of such structures is not proof for arsenic being essential. 

We seem to be preoccupied with controlling energy from dietary intake of protein, fat, and sugar but pay correspondingly very little attention to the intake of essential elements that make metabolism possible, or to the presence of apparently non-essential elements. With modern instrumentation, it has become relatively easy to measure metals with high sensitivity and accuracy. In contrast to the detection of millions of organic compounds to which we are exposed and which generally require quite sophisticated and hence expensive methods of analysis, the number of metals to be screened is less than a hundred and they are relatively inexpensive to monitor. Due to the high reactivity and catalytic potential of metal ions, their levels in our diet and in our environment are an important factor for health, for causing disease or affecting its progression, and for influencing healthy ageing. It is as valid in 2015 as it was discussed in 1951: The functions of trace elements in biology “may hold the answers to many unsolved biochemical and biological problems” [29].
